# Proteomics studies of the interactome of RNA polymerase II C-terminal repeated domain

**DOI:** 10.1186/s13104-015-1569-y

**Published:** 2015-10-29

**Authors:** Gabriel Pineda, Zhouxin Shen, Claudio Ponte de Albuquerque, Eduardo Reynoso, Jeffrey Chen, Chi-Chiang Tu, Wingchung Tang, Steve Briggs, Huilin Zhou, Jean Y. J. Wang

**Affiliations:** Moores Cancer Center, University of California, San Diego, 9500 Gilman Drive, La Jolla, CA 92093 USA; Department of Medicine, Division of Hematology-Oncology, University of California, San Diego, George Palade Laboratories Room 256, 9500 Gilman Drive, La Jolla, CA 92093 USA; Division of Biological Sciences, University of California, San Diego, 9500 Gilman Drive, La Jolla, CA 92093 USA; Ludwig Institute for Cancer Research, University of California, San Diego, 9500 Gilman Drive, La Jolla, CA 92093 USA; Department of Cellular and Molecular Medicine, University of California, San Diego, 9500 Gilman Drive, La Jolla, CA 92093 USA

**Keywords:** ABL, Ionizing radiation, MUDPIT, Phosphotyrosine, pY_1_-CTD antibodies, RPRD1B, SILAC

## Abstract

**Background:**

Eukaryotic RNA polymerase II contains a C-terminal repeated domain (CTD) consisting of 52 consensus heptad repeats of Y_1_S_2_P_3_T_4_S_5_P_6_S_7_ that mediate interactions with many cellular proteins to regulate transcription elongation, RNA processing and chromatin structure. A number of CTD-binding proteins have been identified and the crystal structures of several protein-CTD complexes have demonstrated considerable conformational flexibility of the heptad repeats in those interactions. Furthermore, phosphorylation of the CTD at tyrosine, serine and threonine residues can regulate the CTD-protein interactions. Although the interactions of CTD with specific proteins have been elucidated at the atomic level, the capacity and specificity of the CTD-interactome in mammalian cells is not yet determined.

**Results:**

A proteomic study was conducted to examine the mammalian CTD-interactome. We utilized six synthetic peptides each consisting of four consensus CTD-repeats with different combinations of serine and tyrosine phosphorylation as affinity-probes to pull-down nuclear proteins from HeLa cells. The pull-down fractions were then analyzed by MUDPIT mass spectrometry, which identified 100 proteins with the majority from the phospho-CTD pull-downs. Proteins pulled-down by serine-phosphorylated CTD-peptides included those containing the previously defined CTD-interacting domain (CID). Using SILAC mass spectrometry, we showed that the in vivo interaction of RNA polymerase II with the mammalian CID-containing RPRD1B is disrupted by CID mutation. We also showed that the CID from four mammalian proteins interacted with pS_2_-phosphorylated but not pY_1_pS_2_-doubly phosphorylated CTD-peptides. However, we also found proteins that were preferentially pulled-down by pY_1_pS_2_- or pY_1_pS_5_-doubly phosphorylated CTD-peptides. We prepared an antibody against tyrosine phosphorylated CTD and showed that ionizing radiation (IR) induced a transient increase in CTD tyrosine phosphorylation by immunoblotting. Combining SILAC and IMAC purification of phospho-peptides, we found that IR regulated the phosphorylation at four CTD tyrosine sites in different ways.

**Conclusion:**

Upon phosphorylation, the 52 repeats of the CTD have the capacity to generate a large number of binding sites for cellular proteins. This study confirms previous findings that serine phosphorylation stimulates whereas tyrosine phosphorylation inhibits the protein-binding activity of the CTD. However, tyrosine phosphorylation of the CTD can also stimulate other CTD-protein interactions. The CTD-peptide affinity pull-down method described here can be adopted to survey the mammalian CTD-interactome in various cell types and under different biological conditions.

## Background

The C-terminal repeated domain (CTD) of the largest subunit of RNA polymerase II (RNAPII) consists of heptad repeats with the consensus sequence Y_1_S_2_P_3_T_4_S_5_P_6_S_7_, which is modified by phosphorylation during each transcription cycle to regulate nascent RNA processing and chromatin modifications [1–4]. Previous studies have identified many proteins that specifically interact with S_5_-phosphorylated (pS_5_) or S_2_-phosphorylated (pS_2_) CTD. For example, the 3’-RNA processing factor Pcf11, which contains a CTD-interacting domain (CID) [5], preferentially interacts with pS_2_-CTD [6, 7]; whereas the SRI (Set2 Rbp1 interaction) domain of Set2-histone methyltransferase preferentially interacts with pS_2_pS_5_-doubly phosphorylated CTD [8]. Furthermore, the mammalian capping enzyme Mce1 is activated by its interaction with the pS_5_-CTD [9].

The mammalian RNAPII-CTD is also phosphorylated on Y_1_ [10]. Both ABL1 and ABL2 (ARG) tyrosine kinases can catalyze the stoichiometric phosphorylation of CTD-Y_1_ on RNAPII in vitro [10–14]. Recent phospho-proteomics studies have mapped several tyrosine phosphorylation sites in the mammalian RNAPII-CTD [15, 16], and the yeast RNAPII is also phosphorylated on tyrosine by an unknown kinase [17]. An increase in the levels of RNAPII tyrosine phosphorylation has been observed following DNA damage and correlated with the activation of nuclear ABL tyrosine kinase in mammalian cell lines and mouse tissues [11, 18]. To determine the effect of Y_1_-phosphorylation (pY_1_) on the CTD-protein binding function, we used CTD-peptides as baits to pull-down mammalian cellular proteins and identified these CTD-interacting proteins by mass spectrometry. We used six different CTD peptides, each with four consensus heptad repeats and a unique phosphorylation pattern (no phosphorylation, pY_1_, pS_2_, pS_5_, pY_1_pS_2_, pY_1_pS_5_). We found a number of RNA-binding proteins in the pS_2_- and the pS_5_-peptide pull-down fractions, however, those proteins were not pulled-down by the doubly phosphorylated pY_1_pS_2_-CTD or the pY_1_pS_5_-CTD peptides. The negative effect of pY_1_ on the interaction of pS_2_-CTD with the CTD-interaction domain (CID) was confirmative of a previous report [17]. However, our study also identified proteins that were preferentially pulled-down by the pY_1_pS_2_- or the pY_1_pS_5_-CTD peptide, suggesting that tyrosine phosphorylation can either inhibit or stimulate the protein binding activities of the CTD.

## Methods

### Reagents

Antibodies for Abl 8E9 (BD), H5, and H14 (Covance), c-Myc 9E10, N20, (Santa Cruz), GST (BD), B10 (Millipore), PY20 (Sigma), were used. The biotin-CTD peptides were synthesized by AnaSpec (San Jose, CA). Anti-RPB1 8WG16 monoclonal antibodies were a generous gift from Dr. Richard Burgess, University of Wisconsin-Madison. Polyclonal pTyr_1_-CTD, pTyr_1_pS_2_, pTyr_1_pS_5_ antibodies were generated by Pacific Immunology (Ramona, CA) by injecting 2 rabbits each with either CTSPSpYSPTS peptide, CTSPSpYpSPTS peptide, and CSPTSPpSpYSPT peptide, conjugated at the N-terminus to keyhole limpet hemagglutinin and affinity-purified by binding to the phosphor-peptide-coupled Sepharose beads. Oligonucleotide primers were synthesized by IDT (San Diego, CA). The reactivity of the antibodies was by determined by ELISA against biotin-labeled CTD peptides.

### Plasmids

3XMyc-human SCAF4 CTD interacting domain was generated by ligating PCR products of SCAF4 into KpnI/XhoI digested pcDNA3.0. Three rounds of PCR using were used. The first round used forward primer: 5KpnIMYC15CID (5′-GAC CTA GGT GGG GAA CAG AAA CTG ATT TCG GAA GAA GAT CTC ATG GAC GCC GTC-3′) and reverse primer XhoI15CID (5′-CCG CTC GAG TTA CGC TGC CAT GTC-3′). The second round used was forward primer: 5KpnI2ndMYC: (5′-GAT CTG GGA GGC GAG CAG AAG CTA ATA TCC GAG GAA GAC CTA GGT GGG-3′) and reverse primer: XhoI15CID (5′-CCG CTC GAG TTA CGC TGC CAT GTC-3′). The third round used forward primer: 5KpnI3rdMYC: (5′-GGG GTA CCA TGG AAC AAA AAC TCA TCT CAG AAG AGG ATC TGG GAG GC-3′) and reverse primer: XhoI15CID (5′-CCG CTC GAG TTA CGC TGC CAT GTC-3′). 3XMyc-human full length Mutant RPRD1B was generated by ligating 344 bp DNA fragment-containing mutations synthesized by (GeneScript, N57S, D58S, Q61K, N62R) into KpnI/EcoRI digested RPRD1B pcDNA5.0/FRT. Mutations were generated based on the generous amino acid analysis and modeling of Pcf11 CID to RPRD1B CID by Dr. Dong Wang, University of California, San Diego. 6XMyc-p72 was used unmodified as previously published in [19]. The AblPPn plasmid was generated by two rounds of ligation: in the first ligation, *Sbf*I/*Sal*I digested fragment from CMV-Abl-PP [20] was ligated to PCR product of *Sal*I/μNES*/Xba*I fragment from Abl NES mutant plasmid [21] to generate a *Sbf*I/*Xba*I fragment containing NLS and μNES. In the second round, the *Sbf*I/*Xba*I fragment was further ligated into *Sbf*I/*Xba*I digested CMV-Abl-PP-Nuc. The YF-CTD mutant plasmid used in our studies was pAT7Rpb1(FSPTSPS)_18_ +Cterm Am^r^, which expresses a cDNA of the human Pol II large subunit with a truncated CTD containing 18 peptide repeats that have the tyrosine residue mutated to phenylalanine and a complete CTD C-terminus. This expressed cDNA has an amino-terminal B10 epitope tag and a carboxy-terminal 6XHis tag and was a gift from Dr. David Bentley, University of Colorado—Denver.

### CTD-peptide affinity chromatography

HeLa nuclear extracts were prepared as previously described [22], with the following modifications: the nuclear pellet was sonicated in lysis buffer and spun for 30 min at maximum speed in a table top centrifuge. The supernatant was collected and contained both nucleoplasm and chromatin bound proteins. Lysis buffer consisted of the following: 10 mM HEPES, pH 7.9, 200 mM NaCl, 1.5 mM MgCl2, 0.2 mM EDTA, 0.5 mM DTT, 0.5 % NP40, 0.125 % Sodium deoxycholate, 0.05 % SDS, 10 % glycerol. Before use, 10 mM Na_2_VO_4_, 10 mM β-Glycerophosphate, 1 mM NaF, 1 mM PMSF, and 1X protease cocktail inhibitor (Roche) were added. The nuclear extract was incubated with 4 µg of CTD antibody 8WG16 overnight at 4º C to immunoprecipitate RNAPII using protein A/G beads (Pierce). Prior to immunoprecipitation, the NaCl concentration was adjusted to 400 mM for 30 min. The supernatant, i.e., nuclear extract immunodepleted of RNAPII, was adjusted to 150 mM NaCl. 100 pmols of each of the six different CTD peptides (four consensus repeats with differing phosphorylation’s) were attached to streptavidin-magnetic beads per manufacturer instructions (Roche) and incubated with 5 mg of immunodepleted nuclear extract for 6 h at 4 °C. Beads were washed three times with binding buffer (150 mM NaCl), eluted with SDS-PAGE sample buffer, and fractions were silver stained after running on 4–20 % gel. The eluted fractions were analyzed by mass spectrometry.

### Multidimensional protein identification technology (MUDPIT) mass spectrometry

Proteins were reduced and alkylated using 1 mM Tris (2-carboxyethyl) phosphine (Fisher, AC36383) at 94 °C for 5 min and 2.5 mM iodoacetamide (Fisher, AC12227) at 37 °C in dark for 30 min, respectively. Proteins were digested with 1 μg trypsin (Roche, 03 708 969 001) overnight. Supernatant was collected and centrifuged through a 0.22 μM filter (Fisher# 07-200-386). An Agilent 1100 HPLC system (Agilent Technologies, Santa Clara, CA) delivered a flow rate of 500 nL per minute to a 3-phase capillary chromatography column through a splitter. Using a custom pressure cell, 5 µm Zorbax SB-C18 (Agilent) was packed into fused silica capillary tubing (200 µm ID, 360 µm OD, 20 cm long) to form the first reverse phase column (RP1). A 5 cm long strong cation exchange (SCX) column packed with 5 µm PolySulfoethyl (PolyLC, Inc.) was connected to RP1 using a zero dead volume 1 µm filter (Upchurch, M548) attached to the exit of the RP1 column. A fused silica capillary (100 µm ID, 360 µm OD, 20 cm long) packed with 5 µm Zorbax SB-C18 (Agilent) was connected to SCX as the analytical column (the second reverse phase column). The electro-spray tip of the fused silica tubing was pulled to a sharp tip with the inner diameter smaller than 1 µm using a laser puller (Sutter P-2000). The peptide mixtures were loaded onto the RP1 using the custom pressure cell. Columns were not re-used. The peptide mixtures were loaded onto the RP1 column using the same in-house pressure cell. To avoid sample carry-over and keep good reproducibility, a new set of three columns with the same length was used for each sample. Peptides were first eluted from RP1 column to SCX column using a 0–80 % acetonitrile gradient for 150 min. The peptides were fractionated by the SCX column using a series of 7 step salt gradients (0, 20, 40, 60, 80, 100 mM, and 1 M ammonium acetate for 20 min), followed by high-resolution reverse phase separation using an acetonitrile gradient of 0–80 % for 120 min. The mass spectrometer was operated in positive ion mode with a source temperature of 150 °C and a spray voltage of 1500 V. Data-dependent analysis and gas phase separation were employed. The full MS scan range of 300–2000 *m/z* was divided into 3 smaller scan ranges (300–800, 800–1100, 1100–2000 Da) to improve the dynamic range. Each MS scan was followed by 4 MS/MS scans of the most intense ions from the parent MS scan. A dynamic exclusion of 1 min was used to improve the duty cycle of MS/MS scans. Raw data were extracted and searched using Spectrum Mill (Agilent, version A.03.02). MS/MS spectra with a sequence tag length of 1 or less were considered as poor spectra and discarded. The rest of the MS/MS spectra were searched against the IPI (International Protein Index) database limited to human taxonomy (v3.31, 67,533 protein sequences). The enzyme parameter was limited to full tryptic peptides with a maximum mis-cleavage of 1. All other search parameters were set to SpectrumMill’s default settings (carbamidomethylation of cysteines, ±2.5 Da for precursor ions, ±0.7 Da for fragment ions, and a minimum matched peak intensity of 50 %). Search results for individual spectra were automatically validated using the filtering criteria listed in the following Table.

**Table:** Filtering criteria for autovalidation of database search results

Mode	Protein score	1+ peptide	2+ peptide	3+ peptide
Protein details	>20	>9	>9	>11
Peptide	NA	>13	>13	>15

A concatenated forward-reverse protein database was constructed to calculate the in situ false discovery rate (FDR). The tryptic peptides in the reverse database were compared to the forward database, and were shuffled if they matched to any tryptic peptides from the forward database. The total number of protein sequences in the combined database is 135,069. Proteins that share common peptides were grouped to address the database redundancy issue. The proteins within the same group shared the same set or subset of unique peptides. Only proteins with 2 or more unique peptides were validated. There are 100 proteins observed in the pull-down samples containing CTD peptides (non-modified CTD, pY_1_, pS_2_, pS_5_, pY_1_pS_2_, and pY_1_pS_5_) but not in the beads only control samples. Functional Annotation of these 100 proteins was completed using the Database for Annotation, Visualization and Integrated Discovery (DAVID) [23, 24]. There were no proteins from the reverse database passed the filters mentioned above, which implies the FDR of our protein list is less than 1 %.

### Surface plasmon resonance

Sensograms were recorded on a Biacore T200 instrument using streptavidin (SA) chips. All experiments were conducted at 25 °C and approximately 1000 response units of biotinylated CTD peptides 2.6 nM were immobilized on the chip in a high salt buffer (500 mM NaCl, 10 mM Tris, pH 7.5, 0.5 mM EDTA). Sensograms were run using flow cell 1 (FC 1) as an unmodified reference. Data was collected for FC’s 2, 3 and 4, which contained differentially phosphorylated CTD peptides. FC2 contained unphosphorylated CTD peptide, FC3 contained a CTD peptide only phosphorylated on the serine residue, and FC4 contained a CTD peptide that was phosphorylated on both a serine and tyrosine residue. In all cases 1.2 nM of CID protein was flowed over the chip at 20 μl/min with a 3 min contact time and a 3 min dissociation phase. The running buffer used for the binding experiments was 10 mM Tris, pH 7.5, 150 mM NaCl, 3 mM DTT, and 0.2 mM EDTA. Regeneration was achieved using an 8-min pulse of high salt buffer. Each of the four GST-CID fusion proteins was expressed in BL21 *E.coli* from pDEST™24-CID and purified using glutathione Sepharose (GE Healthcare, Piscataway, NJ) according to manufacturer’s instructions.

### Co-immunoprecipitation of recombinant GST-CTD with Myc-SCAF4-CID

Human embryonic kidney (HEK) 293T cells were cultured in DMEM supplemented with 10 % Fetal Bovine Serum (Hyclone) and 100 µg/ml each of penicillin and streptomycin. 293T cells grown in 10 cm plates to 80 % confluence were transfected with the indicated plasmid using Lipofectamine (InVitrogen) or GeneTran (Biomiga). Cells were harvested in cold PBS, lysed in NETN buffer (20 mM Tris pH 8.0, 100 mM NaCl, 0.5 mM EDTA, 0.5 % Nonident P-40) on ice for 20 min, sonicated and treated with RNase and DNase for 1 h. For co-immunoprecipitation experiments, 250-500 µg of total protein was used for each immunoprecipitation reaction, either with anti-Myc (9E10)-conjugated agarose beads or mouse IgG-coupled A/G Sepharose beads for 1 h at 4 °C.

### Preparation of partially purified RNA polymerase II from HeLa cell nuclear extract

RNA polymerase II was obtained from HeLa cells treated with 8 Gy IR. Following IR treatment HeLa cells were washed once in ice cold 1X PBS and harvested by centrifuging at 1500*g*. The cell pellet was incubated on ice for 10 min in buffer containing 10 mM HEPES, pH 7.9, 100 mM NaCl, 1.5 mM MgCl2, 0.2 mM EDTA, 0.5 mM DTT, 1.0 % Saponin, and 10 % glycerol. Before use, 10 mM Na_2_VO_4_, 10 mM β-Glycerophosphate, 1 mM NaF, 1 mM PMSF, and 1X protease cocktail inhibitor (Roche) were added. After incubation cells were pelleted by centrifugation at 1000*g* and resuspended in phosphate buffer (PB) and layered onto a 30 % sucrose cushion and centrifuged at 1500*g* for 10 min. The pellet (nuclei) was resuspended in 10 mM HEPES, pH 7.9, 150 mM NaCl, 1.5 mM MgCl2, 0.2 mM EDTA, 0.5 mM DTT, 1 % TritonX100, 10 % glycerol, layered on top of 30 % sucrose cushion, and centrifuged at 1500*g* for 10 min. The pellet was washed 3X times with PB and the crude chromatin pellet was extracted using increasing amounts of ammonium sulfate (NH_4_)_2_SO_4_. The supernatant from the 0.5 M fraction contained enriched RNA polymerase II and was used for phosphopeptide mapping.

### Phosphopeptide purification and mapping using immobilized metal affinity chromatography (IMAC)

IMAC was prepared as previously described in [25]. Ni was strip from the resin by 5 mM EDTA, pH8.0, 100 mM NaCl was used to while rotating at room temperature for 1 h in a 50 ml Falcon tube. The stripped resin is pelleted by centrifugation at 1500*g* and washed twice by 50 ml water followed by 50 ml of 0.6 % acetic acid, then, 50 ml of 100 mM FeCl_3_ in 0.3 % acetic acid was used to coordinate iron to NTA resin. Following overnight incubation the resin is washed three times. The first wash was with 50 ml of 0.6 % acetic acid followed by two washes with 50 ml each of 0.1 % acetic acid. After the last wash the volume of the resin is estimated and resuspended in 0.1 % acetic acid as 50 % (vol/vol) slurry and stored at 4 °C. All common chemicals used for IMAC resin preparation where purchased from (SIGMA-Aldrich). SDS was added to the partially purified RNA polymerase II to a final concentration of 1 %. The sample was then reduce and alkylated by with 5 mM DTT for 5 min at 50 °C and 30 mM iodoacetamide for 45 min at room temperature in the dark. Proteins were precipitated by adding 3 volumes of 50 % (vol/vol) ethanol/acetone for 1 h at 4 °C. The protein pellet was resuspended in a buffer composed of 100 mM Tris (pH8.0), 8 M urea. The protein concentration was measured by Bradford assay and 1XTBS was used to dilute the urea concentration to 2 M final. 10 mg of the sample was digested using 0.1 mg of trypsin overnight at 37 °C. Following overnight digest the sample was acidified with TFA to a final concentration of 0.2 % and centrifuged at 4000*g* for 15 min. The soluble peptides were loaded into a 500 mg Sep-Pak18 column and washed twice with 3 ml of 1 % acetic acid, then eluted with a buffer composed of 80 % acetonitrile and 0.1 % acetic acid and dried by speed vac. The dried peptides were resuspended in 100 μl of 1 % acetic acid and loaded to IMAC column containing 70 μl of beads. The IMAC column was washed 2X twice with buffer containing 25 % acetonitrile, 100 mM NaCl, and 0.1 % acetic acid, followed by 1X wash with 1 % acetic acid, and 1X wash with water. The bound peptides were finally eluted with 210 μl of 6 % ammonium and dried by speed vac. Phosphopeptides were resuspended in 80 % acetonitrile and fractionated using a 2 mm Amide-80 column. Fractionated samples were resuspended in 5 μl 1 % TFA, and a 70 min linear gradient from 10 to 40 % ACN and 0.1 % formic acid was used to run the samples into LTQ Orbitrap XL similarly to previously described in [25].

### MS data analysis using SEQUEST

The tandem mass spectra were searched on Sorcerer-sequest system (SageN, San Jose, CA SEQUEST) using a human semi-tryptic IPI database version 3.80 (download from http://www.ebi.ac.uk/IPI). And quantified using XPRESS software from TPP v4.3 rev 1 (Institute for Systems Biology). The search parameters used were: a monoisotopic masses, 50 ppm for the parental mass tolerance, maximum of three modifications per peptide, and a 79.966331 amu variable modification for phosphorylation of serine, threonine, and tyrosine.

### SILAC labeling for identification of CID-dependent interactions with RPRD1B

RPRD1B 293 Flp In cells were grown in conditions used in [26]. Essentially cells were grown in either heavy or light complete DMEM media with 10 % dialyze FBS. Before cells reached 80 % confluence TET induction was initiated for 36 h. After induction of 3XMYC-RPRD1B WT or 3XMYC-RPRD1B MT immunoprecipitation was performed using total cell lysate. Immunoprecipitated RPRD1B was combined reduced, alkylated, and digested by 1 μg of trypsin. Samples were then desalted by 50 mg Sep-Pak18 column and dried by speed Vac. Dried samples were run into a 1 mm amide 80 column, and analyzed by MS as previously described in [25]. The median from the proteins identified with more than three unique peptides was calculated, and proteins with 1.0 cutoff for heavy to light SILAC ratio were determined.

### SILAC labeling for identification of CTD phosphorylation sites affected by ionizing radiation

Three independent SILAC labeling experiments were performed to determine the effect of ionizing radiation (IR) on CTD phosphorylation in HeLa cells. In each experiment, the cells labeled with the heavy amino acids were treated with 8 Gy IR and collected at 2 h after radiation exposure. The cells labeled with the light amino acids were (1) un-irradiated, (2) irradiated with 8 Gy IR and collected 30 min after radiation exposure, or (3) irradiated with 8 Gy IR and collected 60 min after radiation exposure. The lysates from each pair of heavy and light amino acids labeled cells were mixed and RNA polymerase II partially purified as described above. The partially purified RNA polymerase II was then subjected to trypsin digestion and phospho-peptide analysis as described above. The phosphorylation sites in the phospho-containing CTD peptides were quantitated using XPRESS software from TPP v4.3 rev 1 (Institute for Systems Biology) described above.

## Results

### Phospho-CTD-peptide pull-down of cellular proteins

**Fig. 1:**
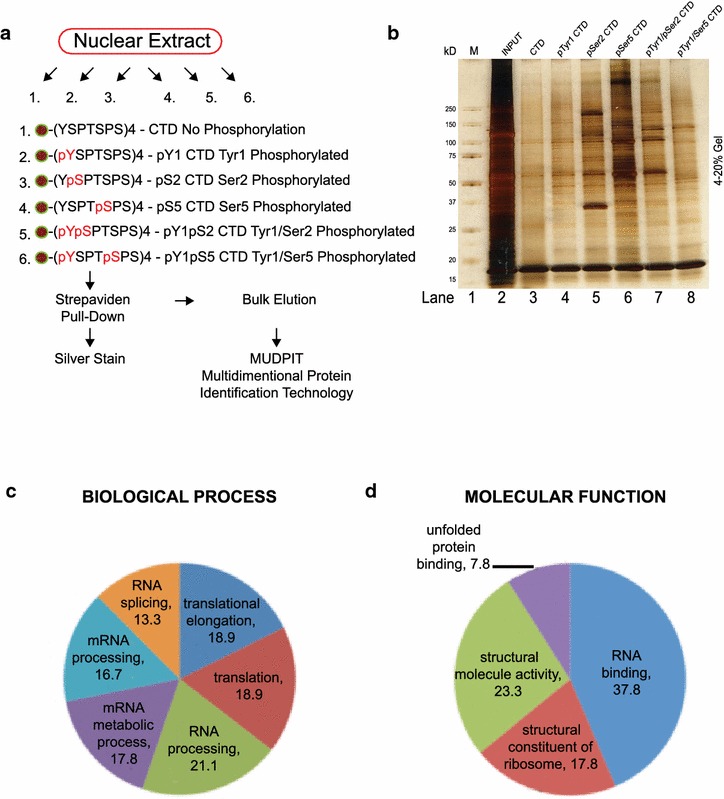
Proteomic analysis of proteins pulled-down by phosphorylated CTD peptides. **a** Summary of experimental strategy. Six CTD-peptides with phosphorylation sites marked in *red* and biotinylation at the N-terminus marked as a *circle* were synthesized and used as affinity probes to pull-down proteins from HeLa nuclear extracts. **b** A representative silver stained gel of proteins pulled-down by the six CTD-peptides. **c**, **d** Graphical representations of bioinformatics analysis of CTD-interacting proteins separated by GO terms in biological process (**c**) or molecular function (**d**). The CTD-interaction proteins are listed in Table 1

A previous study employed biotinylated CTD-peptides with different combinations of serine phosphorylation to investigate the interaction of cellular proteins with the CTD repeats [27]. We adopted this approach to identify CTD-interacting proteins from HeLa nuclear extracts. We synthesized six CTD-peptides (Fig. 1a), each containing four Y_1_S_2_P_3_T_4_S_5_P_6_S_7_ consensus repeats with a biotin at the N-terminus, and each with a different phosphorylation status: (1) unphosphorylated, (2) phosphorylated at the four tyrosines (pY_1_) at the first position, (3) phosphorylated at the four serines at the second position (pS_2_), (4) phosphorylated at the four serines at the fifth position (pS_5_) and (5, 6) combinations thereof (pY_1_pS_2_ and pY_1_pS_5_). HeLa nuclear extracts immunodepleted of the endogenous RNAPII were reacted with each of the six different CTD peptides and bound proteins were identified using multidimensional protein identification technology (MUDPIT). Silver staining displayed the complexity of each of the streptavidin pull-down fractions (Fig. 1b) and showed that the pS_2_ and pS_5_ CTD-peptides pulled-down more proteins than the unphosphorylated or the pY_1_ CTD-peptides (Fig. 1b, compare lanes 5 and 6 to lane 3, 4). Furthermore, the pattern of protein bands pulled-down by the doubly phosphorylated pY_1_pS_2_ or the pY_1_pS_5_ CTD-peptides was dissimilar to that pulled-down by the singularly phosphorylated CTD-peptides (Fig. 1b, compare lanes 7–5, and 8–6). The six pull-down fractions were analyzed by mass spectrometry in two independent experiments. The first by analyses of silvered stained gel bands and the second by MUDPIT analyses of the entire pull-down fraction. A total of 100 proteins were identified from the MUDPIT experiment as summarized in Table 1. Of them, several were also identified by the analysis of gel bands (see proteins marked with ** in Table 1). Some of the proteins in Table 1 are known to directly interact with serine-phosphorylated CTD, e.g., those containing the CTD-interacting domain (CID) (see below). Other proteins pulled-down by the CTD-peptides may represent direct, indirect or non-specific interactions. It cannot be ruled out that these interactions are RNA or DNA dependent, because the nuclear extracts were not treated with nucleases to remove RNA or DNA. A likely example of a non-specific interaction would be GAPDH, an abundant cytosolic protein detected in the pull-down fractions of 4 CTD-peptides (Table 1). However, many other proteins were pulled-down by only one of the six CTD peptides tested (Table 1).

**Table 1:** Proteins Identified by the MUDPIT analyses of CTD-peptide pull-down fractions

Gene symbol	IPI accession number	Percent coverage (%)	Unique peptide	Score	Beads	CTD	tyr1	ser2	ser5	ser2tyr1	ser5tyr1
ESYT2	IPI00409635	38	25	423.67	0	0	0	0	0	46	104
ESYT1	IPI00022143	39	31	497.94	0	0	0	0	0	43	96
PLD1	IPI00218797	29	25	370.49	0	0	0	0	0	17	52
HSP90B1	IPI00027230	24	17	254.42	0	2	0	0	8	5	50
HSPE1	IPI00220362	67	10	134.68	0	3	1	0	7	3	41
CANX	IPI00020984	23	14	196.91	0	0	0	3	0	0	38
PDIA6	IPI00299571	24	9	151.87	0	0	0	0	4	4	34
TRAP1	IPI00030275	14	8	134.8	0	0	0	0	5	0	29
PDIA3	IPI00025252	30	12	176.8	0	0	0	0	4	2	23
P4HB	IPI00010796	24	11	157.3	0	1	0	0	2	1	22
ATP1A1	IPI00006482	13	11	161.54	0	0	0	0	1	0	19
RPL4	IPI00003918	16	6	96.02	0	1	0	7	0	0	19
GAPDH	IPI00219018	24	7	103.11	0	2	0	0	3	6	17
TUFM	IPI00027107	20	7	109.33	0	0	0	0	0	1	16
RPN1	IPI00025874	19	10	117.2	0	0	0	2	0	0	15
LRRC59	IPI00396321	28	7	101.92	0	0	0	0	0	2	15
SFPQ	IPI00010740	11	6	83.98	0	2	0	0	4	1	15
DDX21**	IPI00015953	17	13	174.19	0	0	0	5	0	0	14
LMNA	IPI00021405	16	10	138.09	0	0	0	0	1	0	14
ATP5B	IPI00303476	16	6	97.21	0	0	0	0	0	3	13
KHSRP	IPI00479786	18	9	129.15	0	0	0	0	2	0	12
TUBB	IPI00011654	10	3	51.99	0	0	0	0	0	1	12
CALR	IPI00020599	31	9	123.08	0	0	0	0	2	1	11
ALDOA	IPI00796333	20	5	85.11	0	0	0	0	2	3	11
RAB7A	IPI00016342	26	5	80.8	0	0	0	0	3	0	11
ATP5A1	IPI00440493	14	7	96.43	0	0	0	0	2	2	10
RCN1	IPI00015842	28	7	90.96	0	0	0	0	0	2	10
TKT	IPI00788802	12	6	84.06	0	1	0	0	0	0	10
RPN2	IPI00028635	10	4	61.66	0	1	0	0	0	0	10
RPL7A	IPI00479315	23	7	97.03	0	0	0	3	0	0	9
HNRPU	IPI00644079	10	7	95.98	0	0	0	2	0	1	9
SERBP1	IPI00410693	15	5	79.22	0	0	0	0	1	1	9
SLC25A5	IPI00007188	17	5	68.2	0	0	0	0	0	1	9
RTN4	IPI00021766	2	3	50.99	0	0	0	1	1	0	9
HNRPC**	IPI00477313	26	8	111.38	0	0	0	5	0	0	8
VIM	IPI00418471	21	9	110.44	0	0	0	0	0	2	8
NONO	IPI00304596	16	8	98.5	0	0	0	0	5	0	8
ANXA2	IPI00418169	24	6	93.12	0	0	0	0	0	1	8
MATR3	IPI00789551	12	7	92.94	0	0	0	2	0	0	8
DDX5**	IPI00017617	9	6	79.42	0	0	0	3	0	0	8
PPIB	IPI00646304	25	6	69.98	0	0	0	0	2	0	8
RPL18	IPI00215719	21	4	54.14	0	0	0	4	0	0	8
RPL6	IPI00790342	26	7	105.24	0	0	0	7	0	0	7
CROP	IPI00107745	11	3	52.2	0	0	0	0	0	4	7
RAB1A	IPI00005719	18	3	45.79	0	0	0	0	2	1	7
PRKCSH	IPI00026154	8	3	53.39	0	0	0	0	1	0	6
*RPRD2*	IPI00384541	34	38	543.2	0	0	0	63	0	20	5
PHF3	IPI00170770	17	28	411.08	0	0	0	13	0	83	5
HNRNPUL2	IPI00456887	7	5	67.71	0	0	0	0	0	3	5
RPL7	IPI00472171	13	4	56.17	0	0	0	3	0	0	5
RPL29	IPI00796934	15	3	44.49	0	0	0	1	0	1	5
KIAA1429	IPI00036742	10	17	253.56	0	0	0	14	0	13	4
ZC3H13	IPI00329547	7	12	154.87	0	0	0	7	0	5	4
WTAP	IPI00220302	25	8	120	0	0	0	4	0	3	4
RBMX**	IPI00304692	15	6	75.41	0	0	0	6	0	0	4
HNRPD	IPI00028888	8	3	42.87	0	0	0	0	1	0	4
TMPO	IPI00216230	6	3	40.25	0	0	0	0	1	0	4
SSBP1	IPI00029744	22	2	32.28	0	0	0	0	2	0	4
SFRS3	IPI00010204	14	2	28.23	0	0	0	0	0	0	4
RBM15**	IPI00102752	18	16	217.62	0	0	0	21	0	9	3
NPM1	IPI00549248	17	4	60.16	0	1	0	1	0	1	3
RPL8	IPI00797230	14	4	52.41	0	2	0	4	0	0	3
RPL23A	IPI00789159	16	3	46.69	0	0	0	3	0	0	3
RPL15	IPI00375511	16	4	46.24	0	0	0	10	0	0	3
RPL28	IPI00182533	18	3	37.93	0	0	0	3	0	0	3
LOC654188	IPI00419585	16	3	34.68	0	0	0	0	1	0	3
RPL35	IPI00412607	15	2	31.35	0	0	0	4	0	0	3
RPS14	IPI00026271	15	2	30.42	0	1	0	0	0	0	3
hCG_2040224	IPI00412855	13	2	27.87	0	0	0	2	0	0	3
DST	IPI00074148	0	2	25.88	0	2	1	0	1	1	3
*RPRD1B*	IPI00009659	24	8	121.65	0	0	0	40	0	6	2
RPS9	IPI00221088	20	4	51.03	0	0	0	2	0	0	2
HNRNPH1	IPI00479191	12	4	50.57	0	0	0	0	2	0	2
RPL26	IPI00433834	11	3	35.94	0	0	0	3	0	0	2
SUMO2	IPI00299149	23	2	31.08	0	0	0	0	1	0	2
HNRPM**	IPI00171903	5	4	49.84	0	0	0	3	0	0	1
LOC641293	IPI00845507	18	3	38.98	0	0	0	3	0	0	1
RPL31	IPI00848331	25	3	35.4	0	0	0	2	0	0	1
PRSS1	IPI00011694	9	3	34.11	0	10	39	0	5	13	1
SF3A1	IPI00017451	4	2	31.33	0	1	0	0	0	0	1
RPL10	IPI00646899	8	2	30.62	0	0	0	7	0	0	1
AOC3	IPI00004457	1	2	26.37	0	0	0	0	3	1	1
LOC730004	IPI00787417	23	2	24.8	0	1	0	0	0	0	1
TMPRSS13	IPI00000848	3	2	24.18	0	10	36	0	4	14	1
LACTB	IPI00749059	24	10	145.3	0	0	0	0	0	15	0
*SCAF8*	IPI00744707	7	9	107.99	0	0	0	17	0	0	0
*SCAF4*	IPI00181702	6	7	82.65	0	0	0	12	0	1	0
RNGTT	IPI00000104	7	4	58.94	0	0	0	0	11	0	0
GC	IPI00555812	9	3	43.86	0	3	0	0	0	0	0
DSP	IPI00013933	1	3	42.82	0	3	0	0	0	0	0
FKBP15	IPI00401282	2	3	36.89	0	0	0	0	0	5	0
CBLL1	IPI00290429	5	2	34.71	0	0	0	4	0	0	0
HNRNPUL1**	IPI00013070	3	3	32.92	0	0	0	8	0	0	0
MGAM	IPI00220143	1	2	27.01	0	0	0	0	2	0	0
STOML2	IPI00334190	8	2	23.18	0	0	0	0	0	2	0
KIAA1881	IPI00787298	1	2	22.61	0	0	0	0	2	0	0
PRPF6	IPI00305068	1	2	22.54	0	2	0	0	0	0	0
DIDO1	IPI00619921	1	2	22.43	0	0	0	0	0	2	0
DSG1	IPI00025753	2	2	22.18	0	2	0	0	0	0	0
DSC1	IPI00216099	3	2	20.02	0	2	0	0	0	0	0

Lists the proteins that were identified from peptide pull-down using differentially phosphorylated CTD peptides. The official gene symbol and accession number are listed in the first two columns. Percent coverage column shows the percentage of the protein’s sequence represented by the peptides identified in the MS/MS analysis. Unique peptides column shows total number of peptides that were identified. Score: score from Spectrum Mill. The other columns show the total number of peptides identified in each of the indicated pull-down fractions with beads alone (no peptide), and each of the six CTD peptides illustrated in Fig. 1a. Proteins marked by ** were those identified in two independent experiments by (a) MS/MS identification of gel bands in the pull-down fractions and (b) MUDPIT identification of the entire pull-down fractions. The four CID-containing proteins are highlighted in italics. Note that the MUDPIT experiment identified more proteins, which are listed here

Bioinformatics analysis using Annotation, Visualization and Integrated Discovery (DAVID) of the proteins listed in Table 1 found that the majority of them fall into the Biological Process of RNA splicing and metabolism (Fig. 1c). DAVID also found the Biological Process of translation and the structural constituent of ribosome to be represented (Fig. 1c, d). Given the abundance of ribosomal constituent and the cytoplasmic location of translation, the ribosomal proteins in the pull-down fractions are most likely to be non-specific. On the other hands, Table 1 contains several RNA-binding proteins that are related to known components of the human spliceosomal complexes [28], and those interactions are likely to be relevant because the CTD is known to regulate RNA splicing.

### CID binds pS_2_-CTD but not pY_1_pS_2_-CTD

**Fig. 2:**
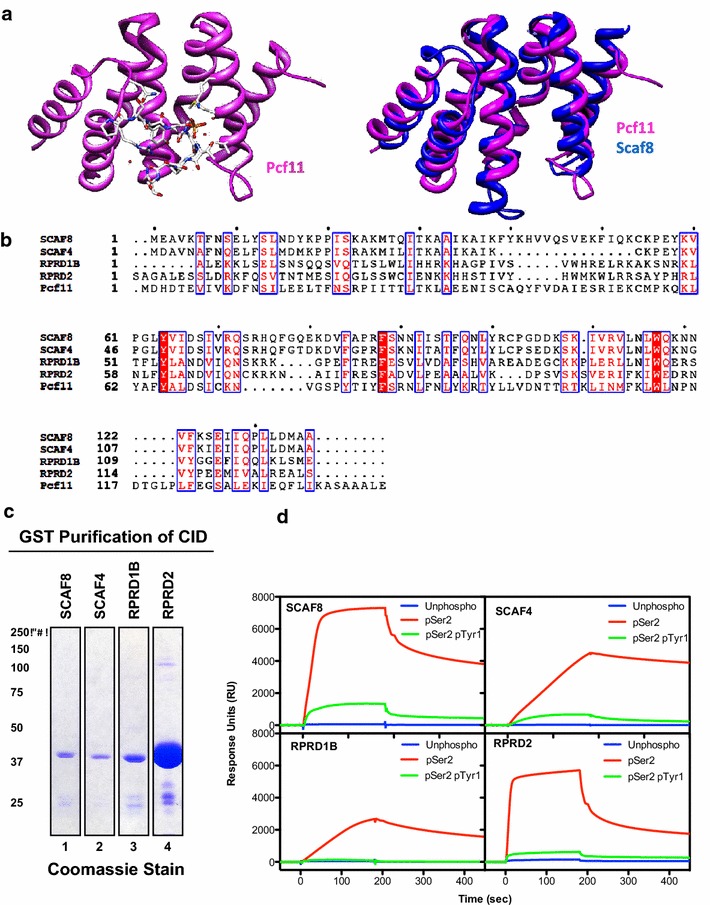
CID binding to pS_2_-CTD but not pY_1_pS_2_-CTD. **a** *Left* crystal structure of the Pcf11 CID domain in complex with a pS_2_-CTD peptide (PDB ID: 1SZA); *Right* superimposition of the CID domain of Pcf11 and SCAF8 (PDB ID: 3D9I). **b** Amino acid alignment of the CID domains in the indicated proteins. **c** GST-CID fusions from each of the indicated CID-containing proteins were purified from bacterial lysates using glutathione affinity chromatography. **d** Binding of the indicated GST-CID to the indicated CTD-peptides was determined by surface plasmon resonance using BIA evalution version 3.1. Unphosphorylated CTD peptide is represented by *blue line*. pS_2_-CTD peptide is represented by *red line*. pY_1_pS_2_-CTD peptide is represented by *green line*

The CTD-interacting domain (CID) was previously identified by a yeast two-hybrid screen for CTD-binding proteins [29]. Subsequent studies have determined that the CID domain of the transcription termination factor Pcf11 interact with phosphorylated serine residues of CTD (pS_2_-CTD) [30], but not with CTD that is doubly phosphorylated on tyrosine and serine (pY_1_pS_2_-CTD) [17]. In Fig. 2a, the complex of Pcf11-CID with pS_2_-CTD is overlaid with the CID of SCAF8 (Fig. 2a) [6, 7, 30, 31]. Although Pcf11 was not among the proteins pulled-down by the pS_2_-CTD peptide, four other CID-proteins, namely SCAF8, SCAF4, RPRD1B, and RPRD2 were identified in the pS_2_-CTD but not in the pY_1_pS_2_-CTD pull-down fractions (Table 1; Fig. 2b). To validate the differential interaction between the CID and the different phosphorylated CTD peptides, we expressed and purified the CIDs from SCAF4, SCAF8, RPRD1B, and RPRD2 as GST-fusion proteins from bacteria (Fig. 2c). Direct interaction between each CID and the biotin-CTD, biotin-pS_2_-CTD and biotin-pY_1_pS_2_-CTD peptides were analyzed by surface plasmon resonance using streptavidin-coated Biacore chips (Fig. 2d) [27, 32]. Consistent with the MUPIT results (Table 1) as well as previously published reports [17, 30], we detected binding of all four CIDs to the pS_2_-CTD peptide but not to the unphosphorylated CTD peptide or the doubly phosphorylated pY_1_pS_2_-CTD peptide (Fig. 2d).

**Fig. 3:**
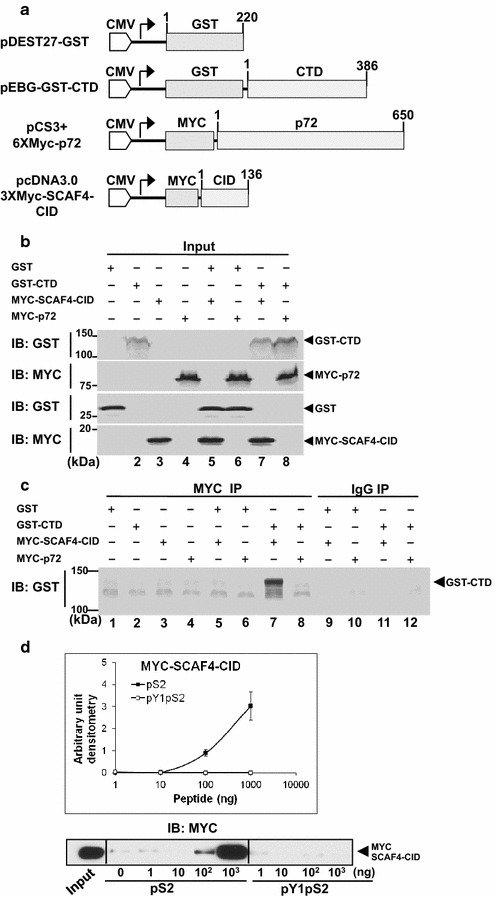
Co-immunoprecipitation of Myc-SCAF4-CID with recombinant CTD. **a** A diagram of mammalian expression constructs expressing GST-CTD and Myc-fusion proteins. **b** Total lysates from HEK293T cells transfected with GST (*lane 1*), GST-CTD (*lane 2*), Myc-SCAF4-CID (*lane 3*), Myc-p72b (*lane 4*) or the indicated combinations (*lanes 5–8*) were immunoblotted with anti-GST or anti-Myc to detect the expression of GST-CTD or the Myc-fusions. **c** Cell lysates as in (**a**) were immunoprecipitated with anti-Myc (9E10) and probed with anti-GST. Total lysates from co-transfected samples (*lanes 5–8*) were also immunoprecipitated with mouse IgG (*lanes 9–12*) and probed with anti-GST. **d** Binding of Myc-SCAF4-CID to biotin-pS_2_-CTD but not biotin-pY_1_pS_2_-CTD peptides. The indicated amount of each biotinylated CTD-peptide was reacted with 50 µg of total lysates from HEK293T cells transfected with the Myc-SCAF4-CID expression plasmid and then pulled down by streptavidin beads. Values shown in the *upper panel* are mean and standard deviation of densitometry quantification of anti-Myc immunoblotting for the Myc-SCAF4-CID in the pull-down fraction from three independent experiments. Representative blots of total lysate (input) and the pull-down fractions are shown in the *lower panel*

We then examined the interaction of the SCAF4-CID with a recombinant GST-CTD protein and with the CTD peptides by immunoprecipitation and pull-down assays (Fig. 3). As shown in Fig. 3b, HEK293T cells were transfected with GST (lane 1), GST-CTD (lane 2), Myc-SCAF4-CID (lane 3), Myc-p72b (lane 4) or combinations (lanes 5–8). Total cell lysates were probed with antibodies for GST or Myc to determine the levels of the transfected proteins. The cell lysates were each reacted with anti-Myc (9E10) or IgG conjugated agarose beads and the precipitated samples were then immunoblotted with anti-GST (Fig. 1c). The results showed that Myc-SCAF4-CID but not Myc-p72b (encoded by DDX17, which is another RNA binding protein involved in RNA processing) interacted with GST-CTD in co-transfected cells (Fig. 3c, lane 7). In Fig. 3d, total lysate from HEK293T cells transfected with the Myc-SCAF4-CID expression plasmid was reacted with pS_2_-CTD and pY_1_pS_2_-CTD peptides over a range of concentrations. The pull-down fractions were then probed with anti-Myc. Densitometry quantification of the immunoblots detecting Myc showed a concentration-dependent interaction of Myc-SCAF4-CID with the pS_2_-CTD peptide but not the pY_1_pS_2_-CTD peptide (Fig. 3d). Together, results shown in Table 1, Figs. 2 and 3 establish that CTD tyrosine-1 phosphorylation disrupts the CID interaction with pS_2_-CTD. These results are consistent with a previous report that Pcf11 interaction with the CTD is disrupted by CTD tyrosine phosphorylation [17].

### CID-dependent interaction of RPRD1B with RNA polymerase II

**Fig. 4:**
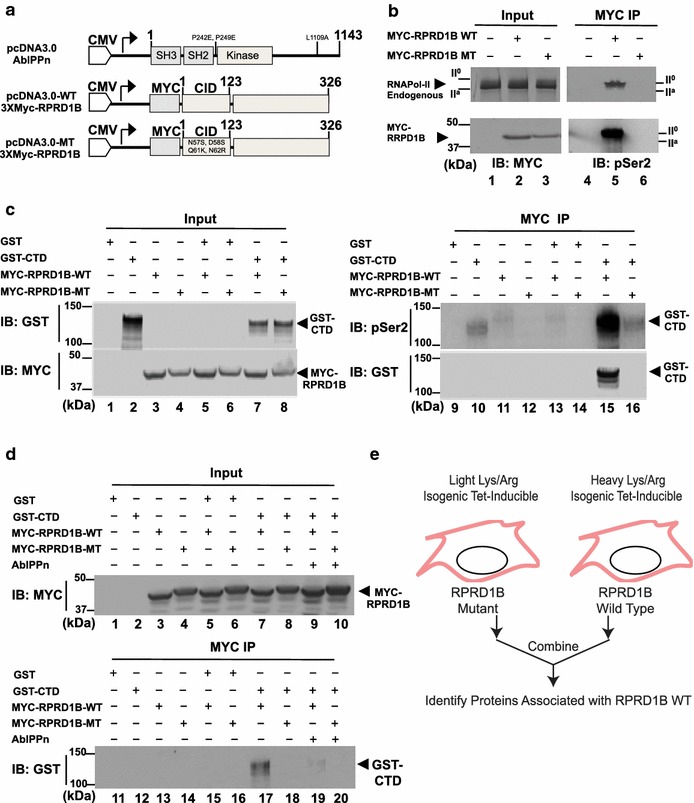
Interaction of full-length Myc-RPRD1B but not CID-mutant Myc-RPRD1B with RNAPII and the CTD. **a** Diagram of full-length wild type (WT) RPRD1B and mutant (MT) RPRD1B containing the following point mutations N57S, D58S, Q61K, N62R in the CID. AblPPn is a constitutively activated Abl kinase in which two proline residues (PP) in the SH2-kinase linker are mutated to glutamic acids to disrupt auto-inhibition, and a leucine residue to inactivate the nuclear export signal (n). **b** WT but not MT RPRD1B interacts with endogenous RNAPII. Total lysates from HEK293T cells transfected vector (*lane 1*), WT Myc-RPRD1B (*lane 2*), MT Myc-RPRD1B (*lane 3*), were immunoblotted with the anti-Myc and anti-8WG16 to detect RPRD1B and RNAPII (*left panels*). These cell lysates were also immunoprecipitated with anti-Myc (9E10) to detect the co-immunoprecipitation of endogenous RNA polymerase II (N20) and pS_2_-CTD (*right panels*). **c** WT but not MT RPRD1B interacts with recombinant CTD. Total lysates from HEK293T cells transfected with GST (*lane 1*), GST-CTD (*lane 2*), or combinations with Myc-RPRD1B (*lanes 3–8*) and probed with anti-GST or anti-Myc (*left panels*, input). These lysates were also immunoprecipitated with anti-Myc (9E10) and probed with anti- pSer2-CTD or anti-GST. **d** C-transfection with AblPPn inhibited RPRD1B interaction with CTD. Total lysates (input) from HEK293T cells transfected with GST (*lane 1*), CTD (*lane 2*), WT RPRD1B (*lane 3*), MT RPRD1B (*lane 4*), or combinations (*lanes 5–8*) including those with AblPPn (*lanes 9, 10*) were probed with anti-Myc (*upper panel*). These ten lysates (*1–10*) were immunoprecipitated with anti-Myc (MYC IP) and the immunoprecipitates probed with anti-GST (*lanes 11–20*, *lower panel*). Note that GST-CTD associated with WT but not MT RPRD1B, and that AblPPn disrupted WT RPRD1B interaction with GST-CTD (compare lane 17–19). **e** Diagram of SILAC mass spectrometry strategy used to identify proteins that associated with WT but not MT RPRD1B. Heavy and light isotope labeling was conducted after tetracyclin-induced expression of the WT and MT RPRD1B in HEK293T cells. See Tables 3 and 4 for summaries of SILAC results

To demonstrate that a mammalian CID containing protein associates with endogenous RNA polymerase II, we generated mutations in the CID domain of the human RPRD1B protein. The mutant RPRD1B contains four amino acid substitutions: N57S, D58S, Q61K, N62R, in its CID domain (Fig. 4a) [7]. To determine whether these CID mutations disrupt RPRD1B interaction with endogenous RNA polymerase II, HEK293T cells were transfected with the wild type or mutant Myc-tagged RPRD1B expression plasmids and the amount of RNAPII or pS_2_-CTD in the anti-Myc (9E10) immunoprecipitates was detected by immunoblotting (Fig. 4b). Immunoblotting of total lysates (Fig. 4b, lanes 1–3) with anti-Myc showed that the wild type (WT) and the CID-mutant (MT) RPRD1B were both expressed in the transfected cells. Following immunoprecipitation with anti-Myc (9E10)-conjugated agarose beads, RNAPII and pS_2_-CTD were co-immunoprecipitated with the WT Myc-RPRD1B, but not the CID-mutated (MT) Myc-RPRD1B (Fig. 4b). We also examined the interaction of the WT and the MT RPRD1B with recombinant GST-CTD. As shown in Fig. 4c, HEK293T cells were transfected with GST (lane 1), GST-CTD (lane 2), WT RPRD1B (lane 3), MT RPRD1B (lane 4) and in combinations (lanes 5–8) the expressed proteins were detected via immunoblotting with anti-Myc and anti-GST in total lysates (input) (Fig. 4c). Following immunoprecipitation using anti-Myc (9E10) conjugated-agarose beads, the precipitated samples were immunoblotted with anti-GST and anti-pS_2_ (Fig. 4c, lanes 9–16). Again, WT RPRD1B associated with pS_2_-CTD (lane 15) but MT RPRD1B did not associate with pS_2_-CTD.

**Fig. 5:**
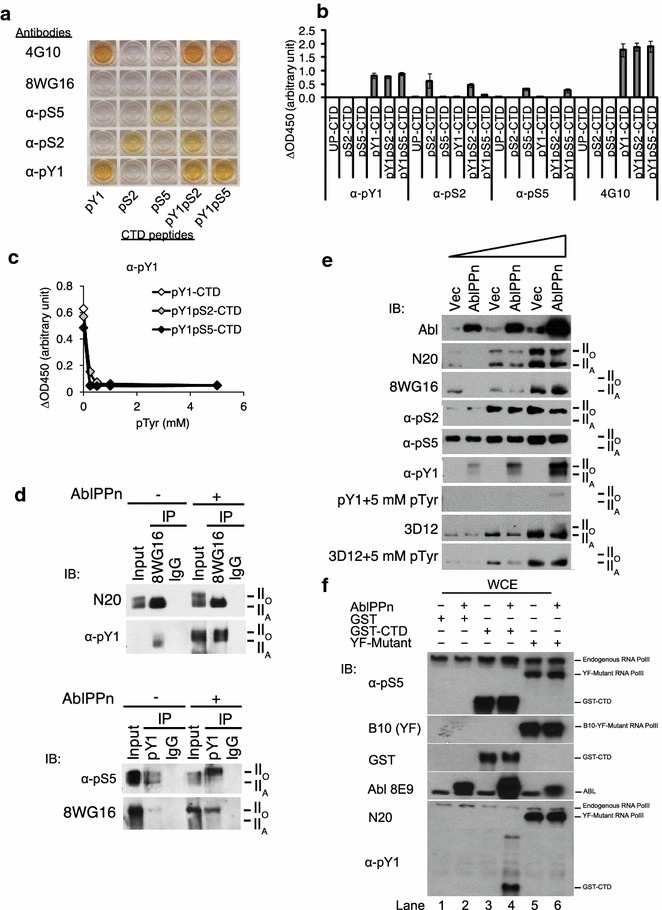
Reactivity of anti-pY_1_-CTD with CTD-peptides and endogenous RNAPII. **a** Representative images of ELISA results. Each *column* is coated with a different CTD peptide as indicated on the *bottom* of the *columns*. Each *row* is reacted with a different antibody as indicated to the *left* of the *rows*. 4G10 is a mouse monoclonal antibody that reacts with phosphor-tyrosine; 8WG16 is a mouse monoclonal antibody that reacts with un-phosphorylated CTD; α-pS_5_ is rabbit polyclonal antibody that reacts with pS_5_ in CTD; α-pS_2_ is a rabbit polyclonal antibody that reacts with pS_2_ in CTD; α-pY_1_ is a rabbit polyclonal antibody raised against a pY_1_pS_5_-CTD peptide. **b** Quantification of ELISA results. Numbers shown are mean ± SD (n = 3). **c** Phosphotyrosine (pTyr) competed with the binding of α-pY_1_ to pY_1_-containing CTD peptides. **d** α-pY_1_-CTD reacts with endogenous RNAPII. HEK293T cells transfected with vector or AblPPn were immunoprecipitated with 8WG16 or IgG and then probed with N20 or anti-pY_1_. Transfection with AblPPn increased the pY_1_-reactivity in whole cell lysate (input) and in 8WG16-precipitated RNAPII (*upper panels*). RNAPII CTD contains 52 CTD-repeats and not all 52 repeats are stoichiometrically phosphorylated in vivo, 8WG16 can react with RNAPII that contains some unphosphorylated CTD repeats and some pY_1_-CTD repeats. In reciprocal immunoprecipitation (IP), whole cell lysates were reacted with anti-pY_1_ and then immunoblotted with anti-pS_5_-CTD or 8WG16. Note that anti-pY_1_ immunoprecipitated RNAPII that reacted with anti-pS_5_-CTD or 8WG16. IIo, RNAPII containing hyper-phosphorylated CTD; IIA, RNAPII with hypo-phosphorylated CTD. **e** The previously reported 3D12 antibody [17] does not react with pY_1_-CTD. Increasing amounts of total lysates from HEK293T cells transfected with vector or AblPPn were immunoblotted with the indicated antibodies. Note that AblPPn did not alter the levels of pS_2_ or pS_5_ reactive RNAPII but increased the levels of pY_1_ reactive RNAPII. The pY_1_-reactivity was competed with phosphotyrosine (pTyr). Note that 3D13 reacts with the IIA form of RNAPII. Reactivity with 3D12 was not affected by the expression of AblPPn. Phosphotyrosine does not inhibit the 3D12 reactivity with RNAPIIA. **f** Anti-pY_1_-CTD does not react with YF-CTD mutant. HEK293T cells were transfected with combinations of AblPPn, GST, GST-CTD or a RNAPII with a truncated CTD in which all of the Y_1_ is mutated to F (phenylalanine). Whole cell lysates were immunoblotted with the indicated antibodies. Note that pS_5_ reacted with the endogenous RNAPII, GST-CTD and the YF-RNAPII. The authenticity of the YF-RNAPII was established by its reactivity with B10 antibody [34]. The YF-RNAPII did not react with anti-pY_1_-CTD

To test if tyrosine phosphorylation of the CTD could disrupt the interaction between CID and CTD, HEK293T cells were co-transfected with GST (lane 1), GST-CTD (lane 2), Myc-RPRD1B WT (lane 3), Myc-RPRD1B MT (lane 4) or combinations with an constitutively activated ABL kinase (AblPPn) (lanes 5–10), and total cell lysates (input) were analyzed via immunoblottings to detect the transfected proteins (Fig. 4d). The AblPPn contains three amino acid substitution mutations to disrupt auto-inhibition and to inhibit nuclear export (Fig. 4a) [21, 33]. These lysates were also subjected to immunoprecipitation using anti-Myc (9E10) conjugated-agarose beads and the precipitates probed with anti-GST (Fig. 4d, lanes 11–20). It was observed that the association between RPRD1B and CTD was disrupted by the co-expression of AblPPn (comparing lane 17–19 in Fig. 4d), correlating with tyrosine phosphorylation of the endogenous RNAPII and GST-CTD (see Fig. 5 below).

We next used SILAC proteomics to identify cellular proteins that differentially associated with the WT vs. the MT RPRD1B in HEK293T cells (Fig. 4e; Table 2). We constructed HEK293 cells to stably express either the WT or the MT RPRD1B from a tetracycline-inducible promoter. Following tetracycline induction, we labeled the WT RPRD1B expressing cells with heavy amino acids, and the MT cells with light amino acids, subjected the labeled lysates to immunoprecipitation with anti-Myc conjugated-beads, and analyzed the resulting immunoprecipitates by tandem mass spectrometry. As summarized in Table 2, 79 proteins were identified to have a median WT/MT ratio of greater than 1.0. It is important to note that the bait protein (RPRD1B) had a median WT/MT ratio of 1.16. The mass spectrometry analysis achieved an over 77 % coverage of RPRD1B in 22 distinct peptides. Bioinformatics analysis of WT-RPRD1B-associated proteins found that the top two biological processes represented were RNA processing (p value 1.1E−29) and mRNA metabolic process (p value 9.7E−27) (Table 3). The top two cellular components represented are ribonucleoprotein complex (p value 2.4E−20) and nucleoplasm (p value 2.0E−17) (Table 3). The top molecular function represented was RNA binding (p value 7.5E−24) (Table 3). As summarized in Table 4, six RNA polymerase II subunits were found to associate with wild type RPRD1B and each with a WT/MT ratio of greater than 2.5, which is significantly above the ratio of the bait RPRD1B protein (Table 4). Over 20 unique peptides were identified as Rpb1, which encodes the largest subunit containing the CTD, with a median WT/MT ratio of 9.0. These results confirmed that the CID domain of RPRD1B is important for its association with the endogenous RNAPII enzyme complex in mammalian cells. Future investigation of the interactions between RPRD1B and the proteins identified in this study (Table 2) will provide clues to the biological function of this mammalian CID-containing protein.

**Table 2:** Summary of SILAC MS analysis of proteins associated with wild-type (WT) vs. CID-mutated (MT) RPRD1B

Gene name	Median of (WT/MT)	Number of unique peptides	Amino acid coverage (%)
POLR2A	9.090909091	20	18.1
POLR2B	7.142857143	17	19.7
RECQL5	4	5	6.2
POLR2C	2.941176471	9	45.8
POLR2D	2.941176471	4	33.8
POLR2E	2.739726027	4	26.2
POLR2I	2.666666667	4	62.4
RPLP2	2.127659574	7	51.3
SKIV2L2	1.5625	3	2.9
NOLC1	1.418439716	4	7.3
EIF2C3	1.408450704	3	17.4
SUPT4H1	1.298701299	3	27.4
SF3A3	1.282051282	3	6.2
DDX3X|DDX3Y	1.265822785	11	32
CSTF3	1.25	5	11.9
TOP3B	1.25	3	9.2
NHP2	1.242236025	6	28.1
DDX5	1.234567901	14	13.3
PARP1	1.234567901	6	9.1
RBM42	1.234567901	4	7.5
RUVBL2	1.234567901	6	12.2
SFRS15	1.234567901	3	2.4
TNRC6B	1.234567901	9	12.6
TFG	1.204819277	5	21
DPF2	1.19760479	8	30.7
PRPF19	1.19047619	5	19.2
CSTF1	1.183431953	6	22.3
DDX5|DDX17	1.183431953	6	30.5
MRPL15	1.176470588	3	18.6
RUVBL1	1.176470588	8	26.8
SNRPD2	1.176470588	10	68.6
SMARCC1	1.169590643	8	20.5
TNRC6C	1.169590643	4	6.2
GTF2I	1.162790698	7	11.9
HMGN2	1.162790698	5	36.7
NUP153	1.162790698	11	10.8
NUP214	1.162790698	9	6.6
RPRD1B	1.156069364	22	77
DDX42	1.149425287	3	3.6
SEC23B	1.149425287	6	15.5
SEC31A	1.149425287	17	18.9
ZNF638	1.149425287	3	5
NUP98	1.142857143	8	7.8
SNRNP70	1.142857143	12	35
DPYSL2	1.136363636	3	13.9
SMARCC2|SMARCC1	1.136363636	11	20.5
DDX3X	1.123595506	3	32
FAU	1.123595506	3	32
SNRPG	1.123595506	3	48.7
RPS7	1.117318436	10	42.6
CDKN2AIP	1.111111111	15	36
CNOT1	1.111111111	3	2.1
EIF2C1	1.111111111	9	21
HNRNPH3	1.111111111	4	15
TP53	1.104972376	6	22.9
PRKRA	1.098901099	3	14.1
PCBP1	1.092896175	4	25
PCF11	1.092896175	6	4.4
FAM98B|FAM98A	1.086956522	3	57.9
FMR1	1.086956522	3	5.5
LRRC59	1.086956522	3	20.5
SF3B3	1.086956522	9	11
DDX17	1.081081081	22	42.4
WDR33	1.081081081	4	5.4
CPSF1	1.075268817	15	14.3
HIST1H1A	1.075268817	3	50.2
SEC24C	1.075268817	16	18.2
CPSF2	1.069518717	10	17.9
HMGA1	1.069518717	4	36.5
DHX9|NPL	1.063829787	3	19.1
EIF2C2	1.063829787	11	30.4
HNRNPD	1.063829787	15	48.4
RBM17	1.063829787	15	20.4
SNRPA	1.063829787	9	36.1
CHERP	1.052631579	8	9.9
DAP3	1.052631579	3	5.8
RPS17	1.052631579	4	34.8
RPS2	1.052631579	3	14.2
SNRPD1	1.052631579	3	58.8

Lists proteins that were identified to preferentially associate with wild type (WT) RPRD1B, but not CID-mutated (MT) RPRD1B utilizing a SILAC mass spectrometry strategy where heavy and light isotope labeling was conducted after tetracycline-induced expression of the WT and MT RPRD1B in HEK293T cells. The official gene symbols of the identified proteins are listed in the first column. The column labeled Median WT/MT is the median ratio between a heavy and light matching peptide identified for that protein. Unique peptides column shows the total number of peptides that were identified and exists in only one protein regardless of peptide length. Amino acid coverage column shows the percentage of the protein’s sequence represented by the peptides identified in the MS analysis

**Table 3:** Functional Annotation of RPRD1B Interacting Proteins

Category	Term	Count	%	P value	Benjamini	FDR
GOTERM_BP_FAT	RNA processing	36	42.9	1.10E−29	4.90E−27	1.60E−26
GOTERM_BP_FAT	mRNA metabolic process	30	35.7	9.70E−27	2.10E−24	1.40E−23
GOTERM_BP_FAT	mRNA processing	28	33.3	1.20E−25	1.80E−23	1.70E−22
GOTERM_BP_FAT	RNA splicing	27	32.1	1.30E−25	1.40E−23	1.80E−22
GOTERM_BP_FAT	RNA splicing, via transesterification reactions with bulged adenosine as nucleophile	22	26.2	2.10E−24	1.80E−22	2.90E−21
GOTERM_BP_FAT	nuclear mRNA splicing, via spliceosome	22	26.2	2.10E−24	1.80E−22	2.90E−21
GOTERM_BP_FAT	RNA splicing, via transesterification reactions	22	26.2	2.10E−24	1.80E−22	2.90E−21
GOTERM_BP_FAT	macromolecular complex assembly	20	23.8	1.40E−09	1.00E−07	2.00E−06
GOTERM_BP_FAT	macromolecular complex subunit organization	20	23.8	4.10E−09	2.60E−07	5.80E−06
GOTERM_BP_FAT	RNA elongation from RNA polymerase II promoter	7	8.3	2.10E−07	1.10E−05	2.90E−04
GOTERM_BP_FAT	mRNA cleavage	5	6	2.50E−07	1.20E−05	3.50E−04
GOTERM_BP_FAT	RNA elongation	7	8.3	3.00E−07	1.30E−05	4.20E−04
GOTERM_BP_FAT	gene silencing by RNA	6	7.1	8.40E−07	3.30E−05	1.20E−03
GOTERM_BP_FAT	chromosome organization	14	16.7	1.70E−06	6.00E−05	2.30E−03
GOTERM_BP_FAT	transcription initiation from RNA polymerase II promoter	7	8.3	1.70E−06	5.60E−05	2.40E−03
GOTERM_BP_FAT	chromatin organization	12	14.3	5.30E−06	1.60E−04	7.40E−03
GOTERM_BP_FAT	chromatin assembly or disassembly	8	9.5	5.30E−06	1.50E−04	7.50E−03
GOTERM_BP_FAT	transcription initiation	7	8.3	5.40E−06	1.50E−04	7.70E−03
GOTERM_CC_FAT	ribonucleoprotein complex	28	33.3	2.40E−20	3.40E−18	2.90E−17
GOTERM_CC_FAT	nucleoplasm	31	36.9	2.00E−17	1.40E−15	2.30E−14
GOTERM_CC_FAT	nuclear lumen	37	44	6.90E−17	5.20E−15	1.30E−13
GOTERM_CC_FAT	intracellular organelle lumen	39	46.4	9.60E−16	3.50E−14	1.20E−12
GOTERM_CC_FAT	organelle lumen	39	46.4	2.00E−15	5.60E−14	2.40E−12
GOTERM_CC_FAT	membrane-enclosed lumen	39	46.4	4.00E−15	9.40E−14	4.70E−12
GOTERM_CC_FAT	nucleoplasm part	19	22.6	5.00E−10	1.00E−08	5.90E−07
GOTERM_CC_FAT	spliceosome	11	13.1	2.00E−09	3.50E−08	2.30E−06
GOTERM_CC_FAT	DNA-directed RNA polymerase II, core complex	6	7.1	7.60E−09	1.20E−07	9.00E−06
GOTERM_CC_FAT	nuclear DNA-directed RNA polymerase complex	6	7.1	1.90E−07	2.70E−06	2.30E−04
GOTERM_CC_FAT	DNA-directed RNA polymerase complex	6	7.1	1.90E−07	2.70E−06	2.30E−04
GOTERM_CC_FAT	RNA polymerase complex	6	7.1	2.40E−07	3.10E−06	2.80E−04
GOTERM_CC_FAT	non-membrane-bounded organelle	33	39.3	9.60E−07	1.10E−05	1.10E−03
GOTERM_CC_FAT	intracellular non-membrane-bounded organelle	33	39.3	9.60E−07	1.10E−05	1.10E−03
GOTERM_CC_FAT	DNA-directed RNA polymerase II, holoenzyme	7	8.3	4.70E−06	5.10E−05	5.50E−03
GOTERM_CC_FAT	chromosome	13	15.5	5.60E−06	5.60E−05	6.60E−03
GOTERM_MF_FAT	RNA binding	35	41.7	7.50E−24	9.00E−22	8.60E−21
GOTERM_MF_FAT	purine NTP-dependent helicase activity	9	10.7	7.10E−08	4.20E−06	8.10E−05
GOTERM_MF_FAT	ATP-dependent helicase activity	9	10.7	7.10E−08	4.20E−06	8.10E−05
GOTERM_MF_FAT	helicase activity	10	11.9	8.20E−08	3.30E−06	9.40E−05
GOTERM_MF_FAT	DNA-directed RNA polymerase activity	6	7.1	4.00E−06	1.20E−04	4.60E−03
GOTERM_MF_FAT	RNA polymerase activity	6	7.1	4.00E−06	1.20E−04	4.60E−03

Summarizes DAVID analysis of RPRD1B interacting proteins listed in Table 1. Gene Ontology terms of biological process (GOTERM_BP), cellular component (GOTERM_CC), and molecular function (GOTERM_MF) are shown. The column designated Count shows the number of genes in the GO term. Percent column shows the number of genes out of the total for each GO term. P-Value, Benjamini, and FDR, are statistical measures to determine confidence of gene enrichment in each GO term

**Table 4:** RNA polymerase subunits identified in RPRD1B interactome

Gene symbol	Median (WT/MT)	Unique peptide	Amino acid coverage (%)	Protein
POLR2A	9.09	20	18.1	POLR2A DNA-directed RNA polymerase II subunit RPB1
POLR2B	7.14	17	19.7	POLR2B DNA-directed RNA polymerase II subunit RPB2
POLR2C	2.94	9	45.8	POLR2C DNA-directed RNA polymerase II subunit RPB3
POLR2D	2.94	4	33.8	POLR2D DNA-directed RNA polymerase II subunit RPB4
POLR2E	2.74	4	26.2	POLR2E DNA-directed RNA polymerased I, II, and III subunit RPABC1
POLR2I	2.67	4	62.4	DNA-directed RNA polymerase II subunit RPB9

Lists RNA polymerase subunits identified to associate with wild type (WT) RPRD1B, but not the CID-mutated (MT) RPRD1B, from the SILAC mass spectrometry experiment where heavy and light isotope labeling was conducted after tetracycline-induced expression of the WT and MT RPRD1B in HEK293T cells. The official gene symbols of the identified proteins are listed in the first column. The column labeled Median WT/MT is the median ratio between a heavy and light matching peptide. Unique peptides column shows the total number of peptides that were identified and exist in only one protein regardless of peptide length. Amino acid coverage column shows the percentage of the protein’s sequence represented by the peptides identified in the MS analysis

### Characterization of antibodies for tyrosine-phosphorylated CTD

Antibodies for serine-2 and serine-5 phosphorylated CTD have been available for many years; however, antibodies for tyrosine-1-phosphorylated CTD were only recently reported [17]. To develop anti-pY_1_-CTD antibodies, we immunized rabbits with three different tyrosine phosphorylated CTD peptides: pY_1_-consensus peptide, pY_1_pS_2_-concensus peptide, and pY_1_pS_5_-consensus peptide and purified phospho-specific antibodies by peptide-affinity chromatography. We found that the pY_1_-consensus peptide generated anti-pY_1_ antibody of low affinity. Immunization with the pY_1_pS_2_-peptide generated antibodies that reacted with pY_1_, pS_2_, pY_1_pS_2_, pS_5_, and pY_1_pS_5_-CTD peptides. However, immunization with pY_1_pS_5_-CTD peptide generated antibody that reacted with pY_1_-CTD, pY_1_pS_2_-CTD and pY_1_pS_5_-CTD but not the serine-only phosphorylated peptides (Fig. 5a, b). This reactivity was competed by phosphotyrosine (Fig. 5c), demonstrating that the antibody recognizes the pY_1_-epitope. The pY_1_-antibody also reacted with endogenous RNA polymerase II in cells transfected with AblPPn (Fig. 5d). We found a significant increase in the reactivity of endogenous RNAPII with our anti-pY_1_ antibody in cells transfected with AblPPn (Fig. 5e). The ectopic expression of AblPPn did not alter the reactivity of RNAPII with the pS_5_- or the pS_2_-CTD antibodies (Fig. 5e). We purchased the previously reported pY1-CTD antibody 3D12 [17]. Despite the report that this antibody reacts with Abl-phosphorylated CTD, we could not repeat that result. As shown in Fig. 5e, the 3D12 antibody reacted with the unphosphorylated RNAPII and its reactivity was not stimulated by the ectopic expression of AblPPn. To further demonstrate the specificity of our pY_1_-CTD antibody, we tested its reactivity against the YF-CTD mutant of RNAPII. As shown in (Fig. 5f), the pY_1_-CTD antibody did not react with the YF-CTD mutant.

### Ionizing radiation alters CTD tyrosine phosphorylation

**Fig. 6:**
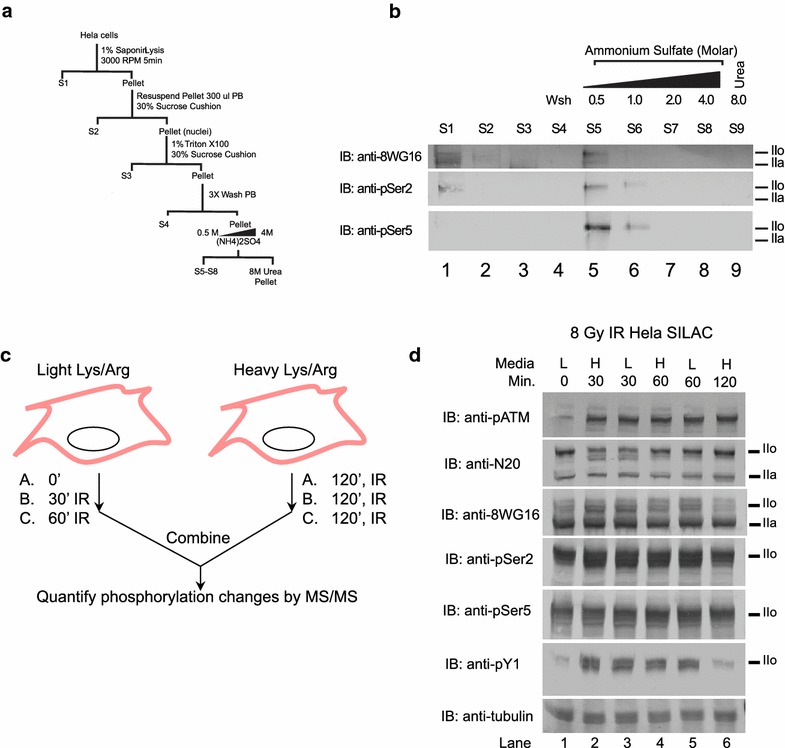
Effect of ionizing radiation on CTD phosphorylation. **a** Purification scheme used to generate partially purified RNAPII from HeLa cell nuclear extract. **b** Detection of RNAPII with three different antibodies in fractions shown in (**a**). **c** Diagram of SILAC mass spectrometry strategy used to examine CTD phosphorylation alterations at 0 h, 30 or 60 min relative to 2 h exposure to 8 Gy of ionizing radiation. **d** RNAPII and CTD phosphorylation in IR-treated cells. Un-fractionated lysates from the indicated HeLa cells at the indicated time (minutes) after 8 Gy of IR were immunoblotted with the indicated antibodies. Note that IR stimulated the phosphorylation of ATM and increased the levels of pY_1_-CTD without changing the levels of pS_2_ or pS_5_ CTD

Previous studies have shown that the nuclear Abl is activated by DNA damage to phosphorylate RNAPII-CTD on tyrosine [11, 18]. We therefore examined IR induced tyrosine phosphorylation of RNA polymerase II CTD using phospho-proteomics combined with SILAC. A multistep purification strategy was established to generate an enriched partially purified fraction of RNA polymerase II that preserved its native phosphorylation state (Fig. 6a). The fractions were characterized using immunoblotting to detect total RNA polymerase II and phosphorylation of serine 2 or serine 5 on CTD (Fig. 6b). SILAC tandem mass spectrometry was then used to compare the CTD phospho-peptides at 2 h after exposure to 8 Gy ionizing radiation (IR) relative to un-irradiated, 30 min irradiated or 60 min irradiated cells (Fig. 6c). As summarized in Table 5, our analysis identified a subset of the previously identified CTD phosphorylation sites, i.e., Y-1874, Y-1881, Y-1909 and Y-1916 that are in the vicinity of the few Lys residues in the CTD. Among this subset of trypsin-released peptides, our SILAC analysis showed that ionizing radiation affected CTD tyrosine phosphorylation in several ways.

**Table 5:** Effect of ionizing radiation on the phosphorylation of RNA polymerase II CTD peptides

Peptide	Phosphorylation site	No IR	IR 2 h	No IR/2 h IR
YSPTSPTphosYSphosPTphosSPK	[1915, 1917, 1919]	2.33E+06	4.43E+06	0.53
YSPTSPTYphosSPTphosSPK	[1916, 1919]	2.79E+06	1.71E+06	1.63
YSPTphosSPTYSphosPTphosSPK	[1912, 1917, 1919]	2.57E+06	3.48E+06	0.74
YSPTSphosPTYSphosPTphosSPK	[1913, 1917, 1919]	3.32E+06	6.08E+06	0.55
YphosSPTSPTYphosSPTTPK	[1874, 1881]	8.42E+05	9.24E+05	0.91
YphosSPTSPTYSphosPTTPK	[1874, 1882]	8.08E+05	5.88E+05	1.37
YSPTSPTYSphosPTphosSPK	[1917, 1919]	4.96E+06	3.16E+06	1.57
YSPTSphosPTYSPTphosSPK	[1913, 1919]	4.76E+06	7.32E+06	0.65
YSPTSPTYSphosPTSphosPK	[1917, 1920]	1.00E+07	6.01E+06	1.66
YSPTSphosPTYSphosPTSphosPK	[1913, 1917, 1920]	3.80E+06	7.93E+06	0.48
YSPTSphosPTYSPTphosSphosPK	[1913, 1919, 1920]	2.33E+06	4.43E+06	0.53
YSPTSphosPTYSphosPTSPK	[1913, 1917]	2.54E+06	7.72E+05	3.29
YSPTSPTYphosSPTSphosPK	[1916, 1920]	4.33E+06	2.87E+06	1.51
YphosSPTSPTYSPTSphosPK	[1909, 1920]	1.40E+06	1.00E+06	1.4
YSPTSPTphosYphosSPTSPK	[1915, 1916]	4.90E+06	2.85E+06	1.72
YSPTphosSPTphosYSPTphosSPK	[1912, 1915, 1919]	1.42E+06	1.97E+06	0.72
YSPTSphosPTYSPTSphosPK	[1913, 1920]	4.76E+06	7.32E+06	0.65
YSPTphosSPTYSphosPTSphosPK	[1912, 1917, 1920]	3.50E+06	4.90E+06	0.71
YSPTSPTphosYSphosPTSphosPK	[1915, 1917, 1920]	2.70E+06	5.23E+06	0.52
YphosSPTSPTYSphosPTSphosPK	[1909, 1917, 1920]	3.32E+06	6.08E+06	0.55

Peptide	Phosphorylation site	30 min IR	2 h IR	30 min IR/2 h IR
YphosSPTSPTYphosSPTTPK	[1874, 1881]	5.91E+05	1.19E+06	0.5
YSphosPTSPTYphosSPTTPK	[1875, 1881]	9.93E+05	1.49E+06	0.67
YSPTphosSPTYphosSphosPTSPK	[1912, 1916, 1917]	2.22E+06	1.22E+06	1.82
YSPTphosSphosPTYSPTphosSPK	[1912, 1913, 1919]	2.23E+05	1.26E+05	1.77
YphosSPTSPTYSphosPTSphosPK	[1909, 1917, 1920]	2.06E+06	3.53E+06	0.58
YphosSPTSPTphosYSPTSphosPK	[1909, 1915, 1920]	2.60E+06	4.02E+06	0.65
YSPTSPTYphosSPTphosSPK	[1916, 1919]	5.65E+06	4.15E+06	1.36
YSPTSPTYSPTphosSphosPK	[1919, 1920]	5.45E+06	4.05E+06	1.35
YSPTphosSPTYSphosPTphosSPK	[1912, 1917, 1919]	2.60E+06	4.02E+06	0.65
YSPTSphosPTYSphosPTphosSPK	[1913, 1917, 1919]	2.60E+06	4.02E+06	0.65
YSPTSPTYphosSphosPTSPK	[1916, 1917]	5.08E+06	3.53E+06	1.44
YSPTSPTYSphosPTphosSPK	[1917, 1919]	5.65E+06	4.15E+06	1.36
YSPTSphosPTYSPTphosSPK	[1913, 1919]	3.14E+06	6.77E+06	0.46
YSPTSPTYSphosPTSphosPK	[1917, 1920]	4.44E+06	3.47E+06	1.28
YSPTSphosPTYSphosPTSphosPK	[1913, 1917, 1920]	9.64E+06	2.73E+07	0.35
YSPTSphosPTYSPTphosSphosPK	[1913, 1919, 1920]	9.64E+06	2.73E+07	0.35
YSPTphosSPTYSPTphosSphosPK	[1912, 1919, 1920]	1.39E+06	1.93E+06	0.72
YphosSPTSphosPTYSPTTPK	[1874, 1878]	7.10E+05	1.48E+06	0.48
YSPTSPTphosYphosSPTSPK	[1915, 1916]	5.08E+06	3.53E+06	1.44
YSphosPTSphosPTYSPTTPK	[1875, 1878]	6.09E+05	1.30E+06	0.47
YSPTSphosPTYSPTSphosPK	[1913, 1920]	4.70E+06	9.61E+06	0.49
YSphosPTSPTYSphosPTSphosPK	[1910, 1917, 1920]	4.08E+06	7.53E+06	0.54
YphosSPTSPTphosYSphosPTSPK	[1909, 1915, 1917]	2.24E+06	1.30E+06	1.72
YSPTphosSPTYSphosPTSphosPK	[1912, 1917, 1920]	3.71E+06	1.01E+07	0.37
YSPTphosSPTYphosSphosPTTPK	[1877, 1881, 1882]	1.69E+06	1.78E+06	0.95
YSPTphosSphosPTYphosSPTSPK	[1912, 1913, 1916]	2.52E+06	1.19E+06	2.12

Peptide	Phosphorylation site	1 h IR	2 h IR	1 h IR/2 h IR
YphosSPTSphosPTYSPTSphosPK	[1909, 1913, 1920]	4.69E+06	1.98E+06	2.37
YphosSPTSPTphosYSPTSphosPK	[1909, 1915, 1920]	2.59E+06	3.55E+06	0.73
YSPTSPTYphosSPTphosSPK	[1916, 1919]	5.08E+06	3.58E+06	1.42
YSPTSphosPTYSphosPTphosSPK	[1913, 1917, 1919]	1.81E+06	2.72E+06	0.67
YphosSPTSPTYphosSPTTPK	[1874, 1881]	6.79E+05	9.01E+05	0.75
YSphosPTSPTYphosSPTTPK	[1875, 1881]	7.12E+05	1.10E+06	0.65
YSPTSPTYSphosPTphosSPK	[1917, 1919]	6.85E+06	4.61E+06	1.49
YSPTSphosPTYSPTphosSPK	[1913, 1919]	3.20E+06	4.32E+06	0.74
YSPTSPTYSphosPTSphosPK	[1917, 1920]	6.85E+06	4.61E+06	1.49
YSPTSphosPTYSphosPTSphosPK	[1913, 1917, 1920]	9.32E+06	2.01E+07	0.46
YSPTSphosPTYSPTphosSphosPK	[1913, 1919, 1920]	3.70E+06	7.89E+06	0.47
YphosSPTSPTYSPTSphosPK	[1909, 1920]	3.78E+06	5.11E+06	0.74
YSPTSphosPTYphosSPTTPK	[1878, 1881]	5.57E+05	2.99E+05	1.86
YSPTphosSPTYphosSPTTPK	[1877, 1881]	7.12E+05	1.10E+06	0.65
YSPTSPTphosYphosSPTSPK	[1915, 1916]	1.27E+06	7.11E+05	1.79
YSPTSphosPTYSPTSphosPK	[1913, 1920]	3.78E+06	5.11E+06	0.74
YSphosPTSPTYSphosPTSphosPK	[1910, 1917, 1920]	1.58E+06	3.75E+06	0.42
YSPTphosSphosPTYphosSPTSPK	[1912, 1913, 1916]	1.30E+06	7.23E+05	1.8
YSPTphosSPTYSphosPTSphosPK	[1912, 1917, 1920]	4.90E+06	1.06E+07	0.46
YSPTphosSPTphosYSPTSphosPK	[1912, 1915, 1920]	4.56E+06	6.41E+06	0.71
YSPTSPTphosYSphosPTSphosPK	[1915, 1917, 1920]	1.61E+06	2.42E+06	0.67

Lists CTD phospho-peptides identified in the SILAC MS analyses of partially purified RNA polymerase II from HeLa cells exposed to ionizing radiation (IR, 8 Gy) for the indicated time. The peptide column lists phosphopeptides identified. Amino acid to the left of “phos” is phosphorylated. The phosphorylation sites in each peptide are listed in the second column. The signals and the ratio from light and heavy peptides are listed in the remaining columns. A ratio >1 represents a reduction in that phospho-peptide at 2 h relative to 0 h, 30 min or 1 h after IR. A ratio <1 represents an increase in that phospho-peptide at 2 h relative to 0 h, 30 min or 1 h after IR

A phospho-peptide containing pY-1874 and pY-1881 but no pS or pT showed similar levels (ratio of 0.91) between non-irradiated and irradiated cells at 2 h, but reduced ratio (0.5) when the comparison was made between cells irradiated for 30 min or 2 h, suggesting that IR caused a transient reduction in pY-1874 and pY-1881 at 30 min with a return to un-irradiated level by 2 h (Table 5). The ratio of 0.75 between 60 min and 2 h irradiated samples was consistent with this transient reduction and recovery of phosphorylation at these two tyrosine sites. A phospho-peptide containing pY-1909 and also pS-1917 and pS-1920 showed reduced levels in un-irradiated and 30 min-irradiated relative to 2 h-irradiated samples (Table 5). This result suggests that IR caused an increase in the abundance of this pY-containing CTD peptide between 30 min to 2 h of IR. A pY-1909, pS-1915 and pS-1920 peptide also showed increased abundance with time from 30 to 60 min relative to 2 h after irradiation (Table 5). Interestingly, a peptide with pY-1909 and pS-1920 was found at higher levels in un-irradiated cells when compared to 2 h-irradiated cells (Table 5). There are two possible interpretations of these results. First, the decrease in pY-1909/pS-1920 peptide may be coupled to the increase in pY-1909/pS-1917/pS-1920 peptide and thus suggesting that IR induced the phosphorylation of pS-1917. Second, the decrease in pY-1909/pS1920 peptide is not related to the increase in pY1909/pS-1917/pS-1920 peptide in that these two phosphorylation configurations occurred on different RNAPII molecules and that IR regulated their levels independently, dependent on the sub-genomic locations of these different RNAPII. With peptides containing the pY-1916 site, our SILAC analyses consistently showed a reduction in abundance at 2-h after IR (Table 5). It thus appears that exposure to ionizing radiation has a complex effect on CTD tyrosine phosphorylation, depending on the phosphorylation site and neighboring pS and pT status. Immunoblotting of total lysates from the HeLa cells used in the SILAC experiment showed a net increase in phospho-ATM up to 2 h after irradiation but a transient net increase in pY_1_-reactivity at 30 and 60 min after irradiation (Fig. 6d). The net increase in pY_1_ levels at 30 and 60 min after IR treatment was likely to have resulted from phosphorylation at other pY_1_ sites that were not detected by the SILAC mapping of tryptic CTD peptides.

## Discussions

Phosphorylation of the CTD generates “codes” for the selective binding of cellular proteins to regulate RNA processing and chromatin structure during transcription elongation [1]. Because each of the 52 repeats of the CTD can be phosphorylated on multiple residues, and because proteins can bind to more than one repeat, the theoretical complexity of the “CTD code” is immense. In this study, we show that synthetic peptides with four heptad repeats of CTD can be used to pull-down mammalian cellular proteins that directly or indirectly interact with the CTD. This approach has identified proteins containing the well-established CTD-interacting domain (CID). This approach also led to the finding that CTD-tyrosine phosphorylation could interfere with the direct binding of CID to pS_2_-CTD consistent with a recently published report that CTD-tyrosine phosphorylation inhibits RNAPII interaction with the Pcf11 transcription termination factor [17]. However, the mass spectrometry analysis has also identified several proteins that interacted with tyrosine/serine doubly phosphorylated CTD peptides.

## Conclusions

While the CTD peptide-pull down method cannot distinguish between direct or indirect binding to alternatively phosphorylated CTD-repeats, it provides a way to survey the proteomic landscape associated with specified combinations of CTD repeat sequences and phosphorylation. This method can also be used to identify proteins that associate with regions of the CTD that contain non-consensus heptad repeats.

## Availability of supporting data

All supporting data have been deposited to the MassIVE repository developed by the NIH-funded UCSD Center for Computational Mass Spectrometry; http://massive.ucsd.edu/ProteoSAFe/status.jsp?task=dfa10c6566dc4bfca6362abc761b74bc

## Abbreviations

CTD: C-terminal repeated domain
RNAPII: RNA polymerase II
CID: CTD-interacting domain
MUDPIT: multidimensional protein identification technology (MUDPIT) mass spectrometry
